# Efficacy Testing of H56 cDNA Tattoo Immunization against Tuberculosis in a Mouse Model

**DOI:** 10.3389/fimmu.2017.01744

**Published:** 2017-12-11

**Authors:** Anouk C. M. Platteel, Natalie E. Nieuwenhuizen, Teresa Domaszewska, Stefanie Schürer, Ulrike Zedler, Volker Brinkmann, Alice J. A. M. Sijts, Stefan H. E. Kaufmann

**Affiliations:** ^1^Department of Infectious Diseases and Immunology, Faculty of Veterinary Medicine, Utrecht University, Utrecht, Netherlands; ^2^Department of Immunology, Max Planck Institute for Infection Biology, Berlin, Germany; ^3^Microscopy Core Facility, Max Planck Institute for Infection Biology, Berlin, Germany

**Keywords:** tuberculosis, vaccine, vaccination, DNA immunization, *Mycobacterium bovis* bacillus Calmette–Guérin, H56

## Abstract

Tuberculosis (TB), caused by *Mycobacterium tuberculosis* (*Mtb*), remains a global threat. The only approved vaccine against TB, *Mycobacterium bovis* bacillus Calmette–Guérin (BCG), provides insufficient protection and, being a live vaccine, can cause disseminated disease in immunocompromised individuals. Previously, we found that intradermal cDNA tattoo immunization with cDNA of tetanus toxoid fragment C domain 1 fused to cDNA of the fusion protein H56, comprising the *Mtb* antigens Ag85B, ESAT-6, and Rv2660c, induced antigen-specific CD8^+^ T cell responses *in vivo*. As cDNA tattoo immunization would be safer than a live vaccine in immunocompromised patients, we tested the protective efficacy of intradermal tattoo immunization against TB with H56 cDNA, as well as with H56_E, a construct optimized for epitope processing in a mouse model. As *Mtb* antigens can be used in combination with BCG to boost immune responses, we also tested the protective efficacy of heterologous prime-boost, using dermal tattoo immunization with H56_E cDNA to boost BCG immunization in mice. Dermal H56 and H56_E cDNA immunization induced H56-specific CD4^+^ and CD8^+^ T cell responses and Ag85B-specific IgG antibodies, but did not reduce bacterial loads, although immunization with H56_E ameliorated lung pathology. Both subcutaneous and intradermal immunization with BCG resulted in broad cellular immune responses, with increased frequencies of CD4^+^ T effector memory cells, T follicular helper cells, and germinal center B cells, and resulted in reduced bacterial loads and lung pathology. Heterologous vaccination with BCG/H56_E cDNA induced increased H56-specific CD4^+^ and CD8^+^ T cell cytokine responses compared to vaccination with BCG alone, and lung pathology was significantly decreased in BCG/H56_E cDNA immunized mice compared to unvaccinated controls. However, bacterial loads were not decreased after heterologous vaccination compared to BCG alone. CD4^+^ T cells responding to Ag85B- and ESAT-6-derived epitopes were predominantly IFN-γ^+^TNF-α^+^ and TNF-α^+^IL-2^+^, respectively. In conclusion, despite inducing appreciable immune responses to Ag85B and ESAT-6, intradermal H56 cDNA tattoo immunization did not substantially enhance the protective effect of BCG under the conditions tested.

## Introduction

Tuberculosis (TB) remains a global health threat, with 10.4 million cases and 1.7 million deaths reported for 2016 (1, 2). It is estimated that approximately a quarter of the world’s population has latent TB infection (LTBI) (3). Socioeconomic factors such as poor living conditions, stress and malnutrition, play a major role in susceptibility to developing TB disease, with the HIV pandemic also a major driver (4). Coinfection with *Mycobacterium tuberculosis* (*Mtb*) and HIV leads to accelerated deterioration of immunity, and TB is the most common cause of death in HIV^+^ individuals. HIV contributes to the increased risk of TB by depleting CD4^+^ T cells, affecting macrophage effector functions, tipping the Th1/Th1 balance and influencing granuloma formation (5). The risk of those with LTBI developing active disease is approximately 10% over a lifetime, but rises to 5–10% per year in those with HIV infection (6). HIV-exposed uninfected infants are another group at high risk of TB infection, as they have poorer T cell generation and IFN-γ production compared to HIV-unexposed infants (7).

An attenuated form of the causative agent of bovine TB, *Mycobacterium bovis* bacillus Calmette–Guérin (BCG), was introduced as a live vaccine in 1921 and is today the most used vaccine globally (8). BCG provides protection against TB meningitis and other forms of disseminated TB. In addition, it has contributed to a reduction in general childhood mortality by boosting non-specific immunity against common causes of childhood disease (9). However, BCG fails to protect adequately against the pulmonary form of TB (10). Protection also varies geographically; in the UK and Norway, BCG confers 50–60% protection that lasts up to 20 years (11), while the high incidence of TB in developing countries illustrates the need for an improved vaccination strategy. Furthermore, BCG can cause severe adverse effects in immunocompromised individuals, and it is not recommended for HIV-infected individuals, who are thus a target group for vaccination due to their increased risk of developing TB (12). As BCG is given at birth throughout the developing world, a new TB vaccine for children should take into account the fact that the majority of the population has already been BCG-vaccinated. New vaccination strategies aim to improve the efficacy and/or safety of BCG in several ways, including modifying it, combining it with booster vaccines, administering it with different adjuvants or altering the route of vaccination (13–19).

A crucial starting point in improving vaccine strategies is knowledge of immune responses that correlate with protection against TB. Although the precise mechanisms of *Mtb* control are unclear, T cell responses are known to be crucial (19). Successful attempts have been made to enhance the cellular response by modifying BCG; for example, a recombinant BCG strain was generated which expresses the *hly* gene encoding for listeriolysin O (LLO) from *Listeria monocytogenes*, in combination with deletion of urease C, resulting in a pH optimal for LLO activity (20–22). Vaccination of mice with BCG *ΔureC::hly* induced more CD4^+^ central memory T cells (T_CM)_ than BCG vaccination, which were protective against infection (21). In other studies, Ag85B-specific CD4^+^ effector memory T cells (T_EM)_ were shown to control infection in the lungs (23), and CD8^+^ T cells also protect against *Mtb*, particularly during LTBI (24–28).

As most individuals in TB-endemic countries are vaccinated with BCG, another strategy for increasing protection against TB is to boost BCG prime immunization with subunit vaccines containing *Mtb* antigens, using non-live vaccines based on recombinant fusion proteins mixed with adjuvants or non-replicating attenuated viral vectors (13, 14, 16, 18, 19). Boosting with a second dose of BCG itself is currently not recommended by the World Health Organization, as it has not been found to improve protection significantly in earlier studies (29–32). Different *Mtb* antigens have been tested against TB, including Ag85A, Ag85B, TB10.4, HBHA, and others (19). Aagaard et al. (33) created the multistage fusion protein vaccine H56, which is composed of three antigens of *Mtb*: Ag85B, ESAT-6, and Rv2660c. Ag85B and ESAT-6 are both immunogenic proteins secreted early in infection (34, 35), with Ag85B found in both *Mtb* and BCG and ESAT-6 only in *Mtb*. Rv2660c was originally identified as a latency-associated gene of *Mtb* (36). Although it has not yet been detected at the mRNA or protein level (37), recombinant Rv2660c stimulated IFNγ responses in CD4^+^ T cells from individuals with LTBI and was also recognized by IgG antibodies (38) and increased protection was found after vaccination of mice with H56 compared to H1, a similar fusion protein that lacks only the Rv2660c gene (35). The Rv2660c (Mb2678c) gene was shown to be expressed at the transcript level by BCG *in vitro* (39). Vaccination with H56 or the antigens of H56 administered with adjuvant CAF01 (40, 41) or IC31 (41, 42) protected against *Mtb* infection in mice. Recently, we showed that intradermal (*i.d*.) tattoo administration of H56 cDNA fused to tetanus toxin fragment C domain 1 (TTFC) cDNA elicited vigorous antigen-specific CD4^+^ T cell and CD8^+^ T cell responses targeted to H56, without the need for adjuvants (43). The immunogenicity of H56 cDNA was enhanced by fusion to TTFC cDNA, and by substitution of the C-terminal epitope flanking residues, to optimize proteasome-mediated epitope generation. Unlike immunization with live bacterial strains such as BCG, cDNA immunization does not carry a risk of infection and it should, thus, be safer for immunocompromised individuals.

In the present study, we aimed to compare the efficacy of *i.d*. DNA tattoo immunization to that of subcutaneous (*s.c*.) BCG immunization *in vivo*, and to test *i.d*. H56 cDNA tattoo immunization as a booster for *s.c*. injection of BCG. We also tested *i.d*. immunization with BCG. Immunization with *i.d*. DNA tattoo alone did not protect against TB. Heterologous vaccination with BCG/DNA improved H56-specific T cell responses, increased antibody titers, and ameliorated lung pathology in our murine TB model. However, bacterial burdens were not reduced when compared to vaccination with BCG alone.

## Materials and Methods

### Mice

Six- to eight-week old CB6F1 mice (male C57BL/6 × female BALB/c F1) were purchased from Charles River. All animal studies were ethically approved by the State Office for Health and Social Services, Berlin, Germany. We designed two protocols, one for single vaccination and one for prime-boost vaccination, each consisting of 10 mice per timepoint divided into two groups of five mice for manageability and performed in two separate experiments.

### DNA and Dermal DNA Tattoo Vaccination

The full-length H56 and H56_E cDNA (33) was codon optimized, and inserted at the 3′end of TTFC cDNA (44, 45) into the pVAX1 vector (Invitrogen) as described previously (43). DNA tattoo immunization was performed with 15 µl cDNA (2 µg/µl) in Tris-EDTA buffer with a 9-needle bar mounted on a tattoo rotary device (Cheyenne) adjusted to 100 Hz at 1 mm depth for 1 min (46), and the mice were under isoflurane anesthesia.

### BCG Vaccination and *Mtb* Challenge

The *Mtb* H37Rv (American Type Culture Collection; catalog no. 27294) and BCG Danish 1331 (BCG SSI) (American Type Culture Collection; catalog no. 35733) were grown in Middlebrook 7H9 broth (BD) supplemented with albumin-dextrose-catalase enrichment (BD), 0.2% glycerol, and 0.05% Tween 80 or on Middlebrook 7H11 agar (BD) containing 10% (vol/vol) oleic acid-albumin-dextrose-catalase enrichment (BD) and 0.2% glycerol. BCG was grown to mid-log phase, washed with phosphate-buffered saline (PBS) and stored at −80°C in PBS/10% glycerol. BCG was washed in PBS before vaccination and administered at a dose of 10^6^ CFU in 100 µl for *s.c*. immunization and 10^6^ CFU in 25 µl for the *i.d*. tattoo. Aerosol challenge with *Mtb* was performed using an inoculum of 20–50 CFU.

### Single Immunization Model

The *s.c*. injection of BCG is known to lead to an approximately 1 log reduction in bacterial burden in mouse models (47, 48) and was included as the standard against which the efficacy of *i.d*. tattoo immunization with BCG or DNA were compared. The DNA vaccines included H56, a DNA vector containing the full-length codon-optimized H56 gene fused to TTFC cDNA, and H56_E, in which six CD8^+^ T cell epitope flanking sequences have been optimized to enhance proteasome-mediated processing (43), and were administered at day 0, 3, and 6. BCG was administered *s.c*. at day 0. One group of mice was also immunized by *i.d*. tattoo with BCG (day 0 only), in order to compare *s.c*. and *i.d*. administration of BCG. Immune responses were measured at day 21 and the efficacy of the methods was tested by subjecting the mice to an aerosol challenge with *Mtb* at day 60.

### Prime-Boost Model

Two groups of mice were *s.c*. immunized with BCG at day 0, and one of these was boosted by *i.d*. H56_E DNA tattoo at day 40 for a heterologous immunization. Another group was primed and boosted *i.d*. with H56_E cDNA tattoo. Unvaccinated mice were included as controls. Immune responses were measured 2 weeks after the booster vaccination. Mice were infected with *Mtb* at day 100 by aerosol challenge and bacterial burdens and lung pathology were assessed at day 190.

### Vaccine-Efficacy Studies

For measurements of the bacterial load, lungs and spleens were harvested and serial dilutions were performed in PBS-0.05% Tween 80 and plated on Middlebrook 7H11 agar. The percentage of inflammation per lung area was measured in a blinded manner on formaldehyde fixed tissue sections stained in Giemsa. Whole lung sections were scanned with a ZEISS Axioscan Z1 driven by ZEN and lung pathology analysis was performed using Volocity (Perkin Elmer). Lung images shown were chosen based on the image analysis, with the lung having the value closest to the average result per group shown here.

### Peptides

All epitopes, as determined previously (43), were synthesized using Fmoc solid phase chemistry. The sequence enumeration of the synthetic peptides referred to the vaccine H56 (33). CD4^+^ T cell epitopes included H56_242–262_ (Ag85B derived) and H56_288–307_ (ESAT-6 derived). The CD8^+^ T cell epitopes included H56_354–363_ (ESAT-6 derived) and five epitopes of Ag85B.

The eighty-five 15mer peptides used as pool to measure the total amount of H56-specific T cells (provided as a kind gift from Dr. Donatella Negri) spanned the entire amino acid sequence of H56, overlapping by 10 amino acid residues, and were synthesized by PRIMM Srl.

### Analysis of Specific CD8^+^ and CD4^+^ T Cell Responses Using Intracellular Cytokine Staining

For intracellular cytokine production, splenocytes were plated overnight with or without 1 µg/ml peptide or peptide pool containing 1 µg/ml of each peptide at 37°C. During the last 4 h, brefeldin (5 µg/ml, Sigma) was added to wells incubated with peptide or phorbol myristate acetate (50 ng/ml, Sigma) and ionomycin (250 ng/ml, Sigma) or to cells incubated with medium alone. Cells were then stained for the cell surface markers named below, fixed with 2% paraformaldehyde, permeabilized with saponin buffer (saponin, 1 g/l; CaCl_2_, 0.11 g/l; MgSO_4_, 0.125 g/l; NaN_3_, 0.5 g/l; bovine serum albumin (BSA) 1 g/l; 10 mM HEPES in PBS, pH 7.4), and stained for intracellular cytokines as described (43). The cell surface- and intracellular cytokine panel consisted of anti-TCRβ-A700 (Biolegend; clone H57-597), anti-CD4 Pacific Blue (Biolegend; clone GK1.5), anti-CD8 PerCP (Biolegend; clone 53-6.7), anti-IL-2 APC (Biolegend; clone JES6-5H4), anti-IFNγ PE-Cy7 (Biolegend; clone XMG1.2), anti-IL-17 PE (Biologend; clone TC11-18H10.1), and anti-TNF-α FITC (clone MP6-XT-22, grown in-house). Samples were acquired on an LSR II cytometer (BD Biosciences) with BD FACS Diva software and analyzed using FlowJo v10 (TreeStar).

### Analysis of Specific CD8^+^ T Cell Responses by IFN-γ ELISpot

Multiscreen ELISPOT plates (Millipore) were coated with 2 µg/ml anti-mouse IFN-γ (clone AN18, grown in-house) in PBS overnight at 4°C. Wells were washed and blocked with 5% BSA/PBS. 5 × 10^5^ erythrocyte depleted lymph node cells were plated with or without 1 µg/ml synthetic peptide overnight in Iscove’s modified Dulbecco’s medium (IMDM) with 10% fetal calf serum (FCS) Pen-Strep at 37°C. Plates were washed with PBS plus 0.01% Tween 20 (PBS-T), and IFN-γ was detected with biotinylated anti-IFN-γ (clone XMG1.2), followed by alkaline–phosphatase (AP)-conjugated streptavidin (homemade) in PBS-T supplemented with 2% BSA. The assay was developed with NBT/BCIP substrate (Thermo Fisher Scientific) and analyzed using an Immunopost S6 Ultra-V Analyzer (Cellular Technology Limited).

### Analysis of T and B Cell Subpopulations

Spleens and inguinal lymph nodes were collected and single-cell suspensions were generated in IMDM 10% FCS Pen-Strep. Cells were surface stained to quantify cell populations. T cell panel: anti-CD3 Alexa 700 (BioLegend; clone 17A2), anti-CD4 PE-Cy7 (BioLegend; clone RM4-5), anti-CD8 V500 (BD Horizon, clone 53-6.7), anti-CD62L APC (BD Pharmingen; clone MEL-14), anti-CD44 Pacific Blue (clone IM7, grown in-house), anti-CXCR5 PE (BioLegend; clone J252D4), and anti-CCR7 PerCP (BioLegend; clone 4B12). B cell panel: anti-B220 PE (BioLegend; clone RA3-6B2), anti-CD38 APC (BioLegend; clone 90), anti-Fas PE-Cy7 (BioLegend; clone Jo2), anti-GL7 FITC (BioLegend; clone GL7), anti-MHCII Pacific Blue (BioLegend; clone M5/114.15.2), anti-CD19 Alexa 700 (BioLegend; clone 6D5), and anti-CD138 Percp (BioLegend; clone 281-2). CD4^+^ T_CM_ were CD3^+^CD4^+^CD44^high^CD62^high^, CD4^+^ T_EM_ were CD3^+^CD4^+^CD44^high^CD62L^low^, T follicular helper cells (T_FH_) were CD3^+^CD4^+^CXCR5^+^, germinal center (GC) B cells were B220^+^CD19^+^GL7^+^Fas^+^, and plasma cells B220^+^CD19^+^CD138^+^CD38^+^.

### Specific Antibody Responses

Mycobacteria-, Ag85B- and ESAT-6-specific antibodies in serum were measured by indirect enzyme-linked immunosorbent assay using *Mtb* H37Rv lysate (BEI Resources), recombinant Ag85B (BEI Resources), or recombinant ESAT-6 (BEI Resources) to coat and AP-labeled anti-mouse IgG, IgG1, and IgG2c for detection (SouthernBiotech).

### Heat Maps

**Figure 1:**
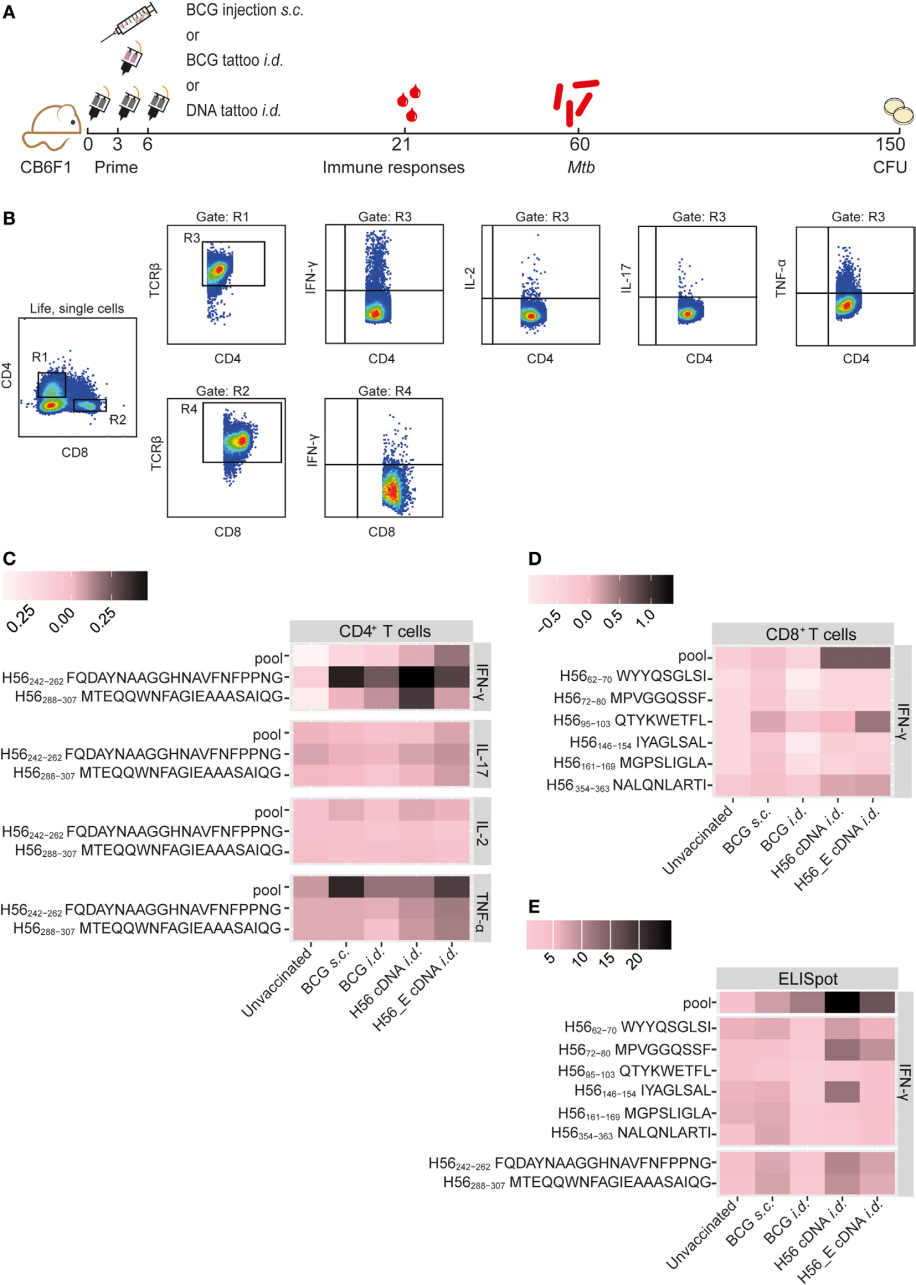
Immunization with H56 or H56_E cDNA induces Ag85B- and ESAT-6-specific T cell responses. Mice were immunized *s.c*. with bacillus Calmette–Guérin (BCG) and *i.d*. with BCG, H56, a DNA vector containing the H56 gene fused to TTFC or H56_E, in which six CD8^+^ T cell epitope flanking residues in the H56 sequence were optimized to enhance proteasome-mediated processing. Intracellular cytokine staining and ELISpots were performed on splenocytes and inguinal lymph node cells, respectively, harvested at day 21 post vaccination and re-stimulated with different H56 peptides. The peptide pools consisted of eighty-five 15-mer peptides spanning the entire sequence of H56, as a measure of the total percentage of H56-antigen-specific cells. CD4^+^ T cell epitopes included H56_242–262_ (Ag85B derived) and H56_288–307_ (ESAT-6 derived). The CD8^+^ T cell epitopes included H56_354–363_ (ESAT-6 derived) and five epitopes of Ag85B. The results show pooled data from two independent experiments (*n* = 10 in total per group). For the unvaccinated group, cells from five mice were pooled into two samples in order to have enough cells. **(A)** Immunization scheme. **(B)** Gating strategy for FACS analysis. After gating on live, single cells, cells were gated on CD4^+^ (R1) or CD8^+^ (R2). Both R1 and R2 were gated on TCR-β^+^ cells (R3) and (R4) before gating single IFN-γ, IL-2, IL-17, and TNF-α cells. **(C)** Heat map showing mean frequency of spleen-derived CD4^+^ T cells secreting IFN-γ, IL-17, IL-2, or TNF-α after peptide stimulation, normalized to medium incubated cells. **(D)** Heat map showing mean frequency of spleen-derived CD8^+^ T cells secreting IFN-γ, IL-17, IL-2, or TNF-α after peptide stimulation, normalized to medium incubated cells. **(E)** Heat map showing mean number of IFN-γ producing cells per million lymph node cells after stimulation with peptide pools, CD4 epitopes or CD8 epitopes, normalized to medium incubated cells. The statistical differences of values shown in the heatmaps are shown in Tables S1 and S2 in Supplementary Material.

The scripts for Figures 1 and 4 are available upon request. FACS data visualization was designed using R-package ggplot2 (v2.1.0; Springer-Verlag New York, NY, USA, 2009.) in R programming language [v3.2.3; R Development Core Team (2008)]. The mean values of percentages of CD4^+^ or CD8^+^ T cells producing cytokines after peptide stimulation were normalized to control samples stimulated with medium and visualized using heat maps. ELISpot data visualization was designed using R-package ggplot2 (v2.1.0; Springer-Verlag New York, NY, USA, 2009) in R programming language [v3.2.3; R Development Core Team (2008)]. The mean numbers of IFN-γ-producing cells per million cells after peptide stimulation were normalized to control samples stimulated with medium. For visualization purposes, the mean value for each experimental condition was transformed to square root of mean response signal and displayed as a heat map.

### Statistics

Data were tested for normality with Levene tests. Kruskal–Wallis with Dunn’s multiple comparison test was used to analyze differences in bacterial burdens between groups. Differences in immunological parameters between groups were analyzed using a one-way ANOVA with Tukey’s multiple comparisons test. *p* < 0.05 was considered significant. The differences in cytokine frequencies were assessed by creating linear regression models with treatment as the predictor and measured cytokine frequency as the dependent variable. The models were created for CD4^+^ cells, CD8^+^ cells, all peptide stimulations, and measured cytokines separately. Benjamini–Hochberg method was used to correct for multiple testing. *p*-values <0.05 were considered significant.

## Results

### Immunization with H56 or H56_E cDNA Induces H56-Specific T Cell Responses

Mice were immunized with BCG (*s.c*. or *i.d*.), with H56, a DNA vector containing the full-length codon-optimized H56 gene fused to TTFC cDNA, or with H56_E, in which six CD8^+^ T cell epitope flanking residues in the H56 sequence were optimized to enhance proteasome-mediated processing (43) (Figure 1A). Previously, we identified seven novel CD8^+^ T cell epitopes in the H56 fusion protein (43). In order to compare Ag85B- and ESAT-6-specific T cell responses induced by the cDNA vaccines and BCG, we measured peptide-specific immune responses after the different immunization strategies, including responses to an H56 peptide pool. Splenocytes or lymph node cells from vaccinated mice were re-stimulated *ex vivo* with Ag85B- and ESAT-6-derived CD8^+^ (43) and CD4^+^ (49, 50) T cell epitopes and responses in the spleen were measured using intracellular cytokine staining and flow cytometry (Figures 1B–D) or in the draining lymph nodes by IFN-γ ELISpot (Figure 1E). The mean percentages of cytokine-producing CD4^+^ or CD8^+^ T cells after peptide stimulation were normalized to control samples stimulated with medium and visualized using heat maps (Figures 1C,D). Results of statistical analysis are shown in Table S1 in Supplementary Material. Incubation of splenocytes with a pool of overlapping 15-mers covering the entire H56 sequence induced increased IFN-γ production by CD4^+^ T cells from H56 and H56_E *i.d*. vaccinated mice compared to unvaccinated controls (Figure 1C). H56 cDNA immunized mice had increased IFN-γ production in response to both the Ag85B-derived peptide H56_242–262_ and the ESAT-6-derived peptide H56_288–307_. No differences in TNF-α, IL-17, or IL-2 secretion by spleen CD4^+^ T cells were detected between the groups (Figure 1C). Differences in CD8^+^ T cell IFN-γ production were not significant, although there was a trend toward increased recognition of the H56 protein (as measured by responses to the peptide pool) in the H56 or H56_E cDNA *i.d*. immunized mice (Figure 1D). IFN-γ ELISpot analysis of total lymph node cells also demonstrated increased numbers of cells producing IFN-γ in the H56 cDNA *i.d*. vaccinated mice after stimulation with the H56 peptide pool compared to both unvaccinated and BCG *s.c*. vaccinated mice (Figure 1E; Table S2 in Supplementary Material). Overall, the H56 cDNA tattoo immunization increased H56-specific IFN-γ-producing T cell responses.

### Increased CD4^+^ T_EM_, T_FH_, and GC B Cells following Vaccination with BCG

**Figure 2:**
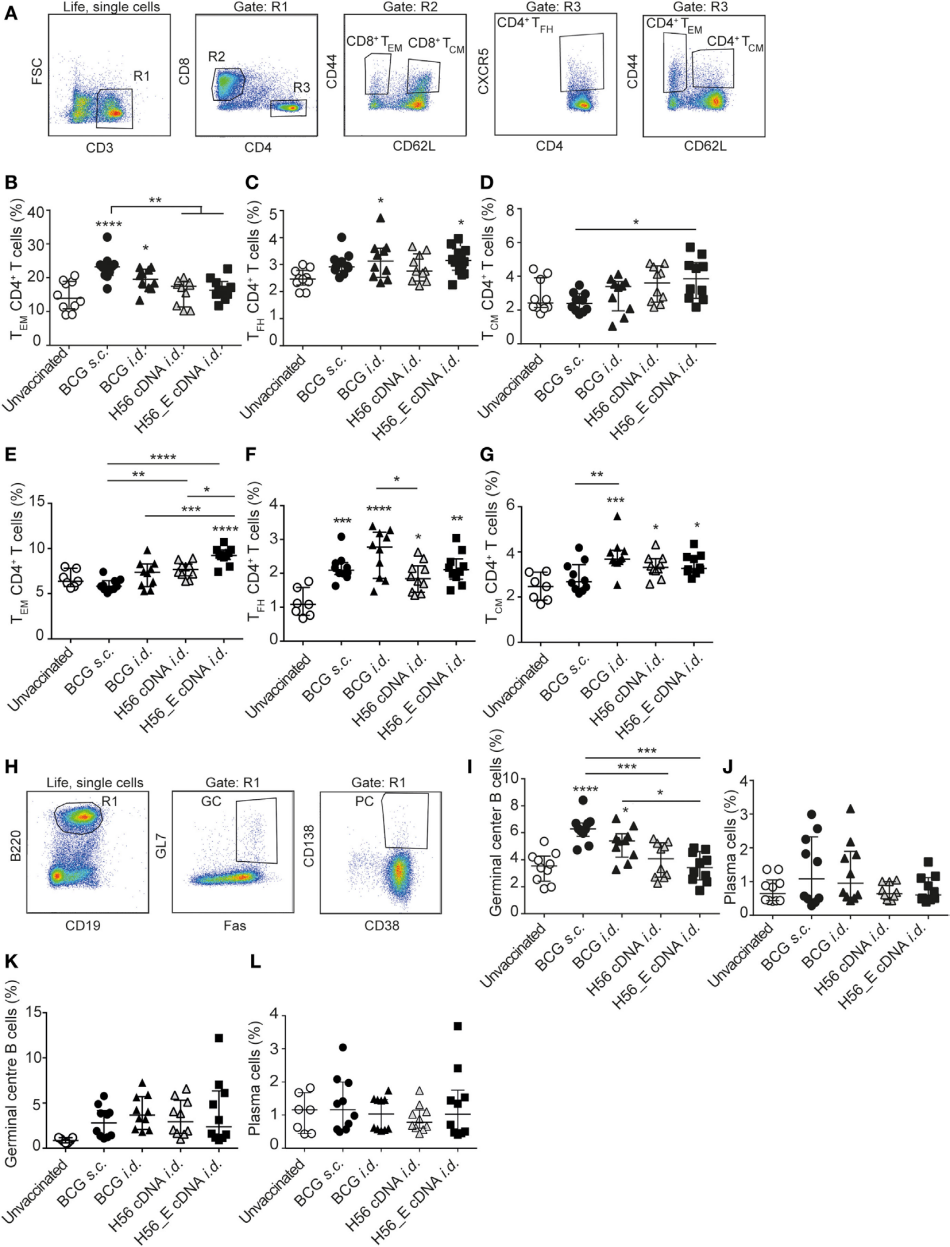
Differences in T- and B cell sub-populations following immunization with cDNA and BCG. Samples were collected at day 21 post vaccination. Splenocytes and lymph node cells were stained for different surface markers and analyzed by FACS. Pooled data of two experiments (*n* = 10 in total per group) are shown ±SEM. **(A)** Gating strategy for FACS analysis for T cell sub-populations. After gating on live, single cells, cells were gated on CD3^+^ (R1). R1 was gated on CD8^+^ (R2) or CD4^+^ (R3). CD4^+^ and CD8^+^ T cells were then analyzed for expression of CD44, CD62L, and CXCR-5 to identify CD4^+^ T_CM_, CD4^+^ T_EM_, and CD4^+^ T_FH_ subsets. **(B–D)** Percentages of **(B)** T_EM_, **(C)** T_FH_, and **(D)** T_CM_, among spleen CD4^+^ T cells. **(E–G)** Percentages of **(E)** T_EM_ **(F)**, T_FH_, and **(G)** T_CM_, among lymph node CD4^+^ T cells. **(H)** Gating strategy for FACS analysis for B cell sub-populations. After gating on live, single cells, B cells were gated as B220^+^ and CD19^+^ (R1). Expression of GL7, Fas, CD138 and CD38 to identify plasma cells and germinal center (GC) B cells. **(I)** Percentages of GC B cells among spleen B cells. **(J)** Percentages of plasma cells among spleen B cells. **(K)** Percentage of GC B cells among lymph node B cells. **(L)** Percentage plasma cells among lymph node B cells. **(A–L)** Significant differences between groups compared to the unvaccinated group are marked as * and significant differences between the groups with lines (ANOVA with Tukey’s multiple comparison test; *p < 0.05; **p < 0.01; ***p < 0.001; ****p < 0.0001).

T cell memory populations and B cell responses after vaccination were measured by flow cytometry at day 21 in spleens and draining lymph nodes (Figure 2A). Both *s.c*. and *i.d*. BCG immunization resulted in increased percentages of CD4^+^ T_EM_ among spleen CD4^+^ T cells compared to unvaccinated mice (Figure 2B), with CD4^+^ T_FH_ showing similar trends (Figure 2C). Frequencies of CD4^+^ T_CM_ were higher in the spleens of DNA *i.d*. vaccinated mice than in BCG *s.c*. vaccinated mice (Figure 2D). T_EM_ responses differed in draining lymph nodes in that only H56_E immunized mice had increased T_EM_ (Figure 2E). A similar pattern was observed for T_FH_ in lymph nodes compared to spleen, with all responses increased in all vaccinated groups compared to unvaccinated controls (Figure 2F). This pattern was also seen for T_CM_ in draining lymph nodes (Figure 2G). There were no significant differences in frequencies of CD8^+^ T_EM_ or CD8^+^ T_CM_ between the groups (data not shown).

**Figure 3:**
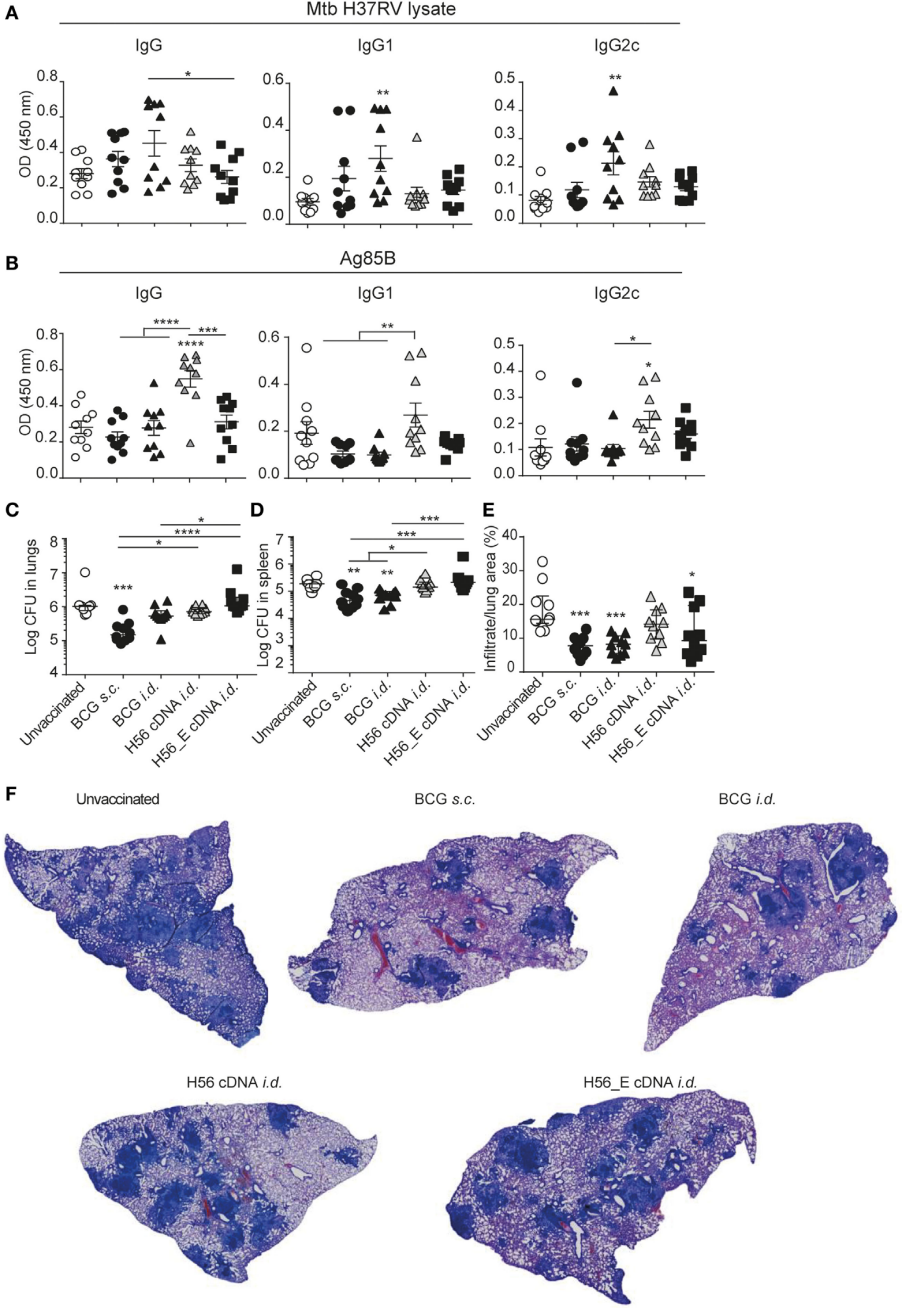
H56 or H56_E cDNA tattoo is not protective against *Mycobacterium tuberculosis* (*Mtb*) infection in mice. Serum samples were collected at day 21 post vaccination and analyzed by enzyme-linked immunosorbent assay. Lungs and spleens were collected at day 90 post *Mtb* challenge and plated in serial dilution. Pooled data of two experiments with (*n* = 10 in total per group) are shown ±SEM. **(A)** *Mtb* lysate specific total IgG, IgG1, and IgG2c antibody titers as measured at 1:10 dilution. **(B)** Ag85B lysate specific total IgG, IgG1, and IgG2c antibody titers as measured at 1:10 dilution. **(C)** Bacterial loads in lungs and **(D)** spleen. **(E)** Quantification of percentage of infiltrated area per lung. **(F)** Giemsa staining of infected lungs. Representative histology slides per group are shown. **(A,B,E)** Significant differences between antibody titers of treatment groups compared to the unvaccinated group are marked as * and significant differences between the groups with lines (ANOVA with Tukey’s multiple comparison test; *p < 0.05; **p < 0.01; ***p < 0.001; ****p < 0.0001). **(C,D)** Significant differences between the groups are shown with * (Kruskal–Wallis with Dunn’s multiple comparison test; *p < 0.05; **p < 0.01; ***p < 0.001).

Humoral responses may contribute to protection against TB (51–53). We measured the frequencies of plasma cells and GC cells among B cells by flow cytometry (Figure 2H) in spleens (Figures 2I,J) and draining lymph nodes (Figures 2K,L). Frequencies of GC B cells were increased in spleens of both groups vaccinated with BCG compared to unvaccinated mice (Figure 2I), with a similar trend in plasma cells (Figure 2J). In the draining lymph nodes, GC B cells appeared increased in all immunized groups (Figure 2K) while plasma cells were similar between groups (Figure 2L). Antibody responses were very variable between animals, possibly because of the mixed background of CB6F1 mice, which are first-generation offspring of female BALB/c and male C57/BL6 mice. Although antibody responses varied between mice, the trend was directed toward increased levels of mycobacteria-specific IgG, including IgG1 and IgG2c, in mice vaccinated with BCG, which expresses multiple mycobacterial proteins (Figure 3A) and increased levels of Ag85B-specific IgG, IgG1, and IgG2c in the H56 cDNA vaccinated group (Figure 3B). ESAT-6-specific antibodies were not detected (data not shown). Curiously, antibody levels were lower in mice immunized with H56_E DNA than with H56 DNA, suggesting that B cell epitopes may have been influenced by the sequence modification.

### H56 and H56_E cDNA Tattoo Immunization Does Not Reduce Bacterial Loads in Mice

To evaluate the protective efficacy of the different vaccines, bacterial loads were measured in lungs and spleens at day 90 after infection with *Mtb* and lung pathology was quantified as cell infiltration per lung area in a blinded manner. Mice vaccinated with BCG *s.c*. benefited from an approximately 1 log reduction in bacterial load in the lung compared to unvaccinated mice, whereas mice vaccinated with BCG *i.d*. did not (Figure 3C). Bacterial loads were not decreased after *i.d*. immunization with H56 or H56_E cDNA. A similar trend was seen in the spleen (Figure 3D). Furthermore, mice vaccinated with BCG strains had strongly decreased lung inflammation compared to unvaccinated mice (Figures 3E,F). H56 cDNA immunization did not decrease lung pathology compared to unvaccinated mice; however, there was reduced lung pathology in H56_E cDNA immunized mice (Figures 3E,F). Overall, cDNA tattoo immunization *i.d*. with H56 constructs was not as protective as BCG *s.c*. against TB in our model, but H56_E immunization ameliorated pathology.

### Boosting BCG with *i.d*. H56 cDNA Tattoo Immunization Increases Ag85B and ESAT-6 Specific CD4^+^ and CD8^+^ T Cell Cytokine Responses

**Figure 4:**
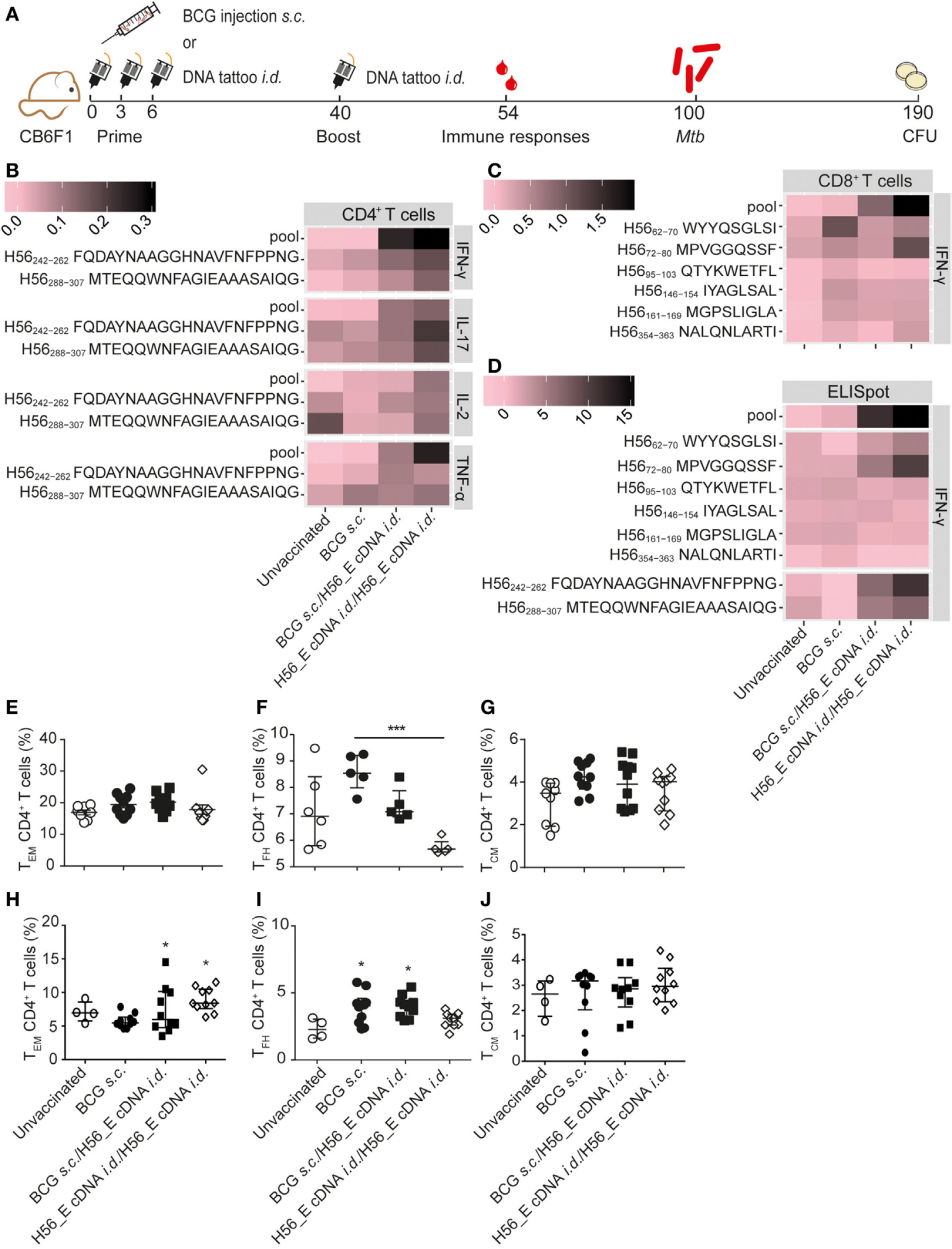
Boosting bacillus Calmette–Guérin (BCG) with H56_E cDNA tattoo immunization increases H56-specific cytokine-producing cells. Intracellular cytokine staining and ELISpot was performed on splenocytes and inguinal lymph node cells, respectively, harvested 2 weeks after the booster vaccination and re-stimulated with different H56 peptides. The peptide pools consisted of eighty-five 15-mer peptides spanning the entire sequence of H56 as a measure of the total percentage of H56-antigen-specific cells. CD4^+^ T cell epitopes included H56_242–262_ (Ag85B derived) and H56_288–307_ (ESAT-6 derived). The CD8^+^ T cell epitopes included H56_354–363_ (ESAT-6 derived) and five epitopes of the Ag85B portion. For the unvaccinated group, cells from five mice were pooled into two samples in order to have enough cells. Splenocytes and lymph node cells were also stained for different surface markers and analyzed by FACS. Gating strategy is shown in Figure 2A. The results show pooled data from two independent experiments (*n* = 10 in total per group). **(A)** Immunization scheme. **(B,C)** Heat maps showing mean frequency of **(B)** CD4^+^ T cells secreting IFN-γ, IL-17, IL-2, or TNF-α after peptide stimulation, normalized to medium incubated cells and **(C)** CD8^+^ T cells secreting IFN-γ after peptide stimulation, normalized to medium incubated cells. **(D)** Heat map showing mean number of IFN-γ-producing cells per million lymph node cells after stimulation with peptide pools, CD4 epitopes or CD8 epitopes, normalized to medium incubated cells. The statistical differences of the values shown in the heatmaps are listed in Tables S3 and S4 in Supplementary Material. **(E–G)** Percentages of **(E)** T_EM_, **(F)** T_FH_, and **(G)** T_EM_, among spleen CD4^+^ T cells. **(H–J)** Percentages of **(H)** T_EM_, **(I)** T_FH_, and **(J)** T_EM_ among lymph node CD4^+^ T cells. **(E–J)** Significant differences compared to the unvaccinated group are marked as * and significant differences between the groups with lines (ANOVA with Tukey’s multiple comparison test; *p < 0.05; **p < 0.01; ***p < 0.001).

Although *i.d*. H56_E DNA tattoo vaccination did not reduce *Mtb* burdens, it ameliorated lung pathology, and we hypothesized that it may increase the efficacy of the BCG vaccine in a prime-boost system by boosting responses to Ag85B and inducing responses to the *Mtb-*specific antigens ESAT-6 and Rv2660c found in H56. Therefore, we tested a heterologous prime-boost vaccination regimen (Figure 4A). In addition, we tested whether repeating *i.d*. H56_E DNA tattoo immunization (homologous prime/boost) could improve its protective efficacy. Spleen H56-specific CD4^+^ IFN-γ responses were increased at day 14 after boosting of BCG with *i.d*. H56 and H56_E DNA tattoo immunization as well as by homologous H56_E cDNA immunization, compared to BCG-only immunized groups (Figure 4B; Table S3 in Supplementary Material). In addition, spleen H56-specific CD4^+^ TNF-α responses were increased in the homologous H56_E cDNA immunized group compared to the BCG *s.c*. immunized group, and in both groups boosted with H56_E DNA compared to the unvaccinated controls. The BCG/H56_E immunized group had increased frequencies of Ag85B-specific CD4^+^TNF-α^+^ cells. Homologous cDNA prime-boost induced increased percentages of H56-specific and ESAT-6-specific IFN-γ CD8^+^ T cells compared to both BCG *s.c*. immunized and unvaccinated groups (Figure 4C; Table S3 in Supplementary Material). BCG *s.c*. immunized mice had increased Ag85B-specific CD8^+^IFN-γ^+^ responses compared to unvaccinated mice, which were further boosted by H56_E cDNA compared to BCG alone. ELIspots on lymph node cells showed increased IFN-γ responses to Ag85B and ESAT-6 CD4 epitopes as well as the H56 peptide pool in mice that had received homologous H56_E cDNA prime-boost (Figure 3D; Table S4 in Supplementary Material). Analysis of T cell subsets demonstrated no differences in frequencies of spleen T_EM_, T_CM_, or T_FH_ after DNA boosting (Figures 4E–G), with a similar pattern in the draining lymph nodes (Figures 4H–J). Frequencies of CD8^+^ T_CM_ and CD8^+^ T_EM_ were not significantly different after different immunization strategies, similar to the single vaccination experiments (data not shown).

**Figure 5:**
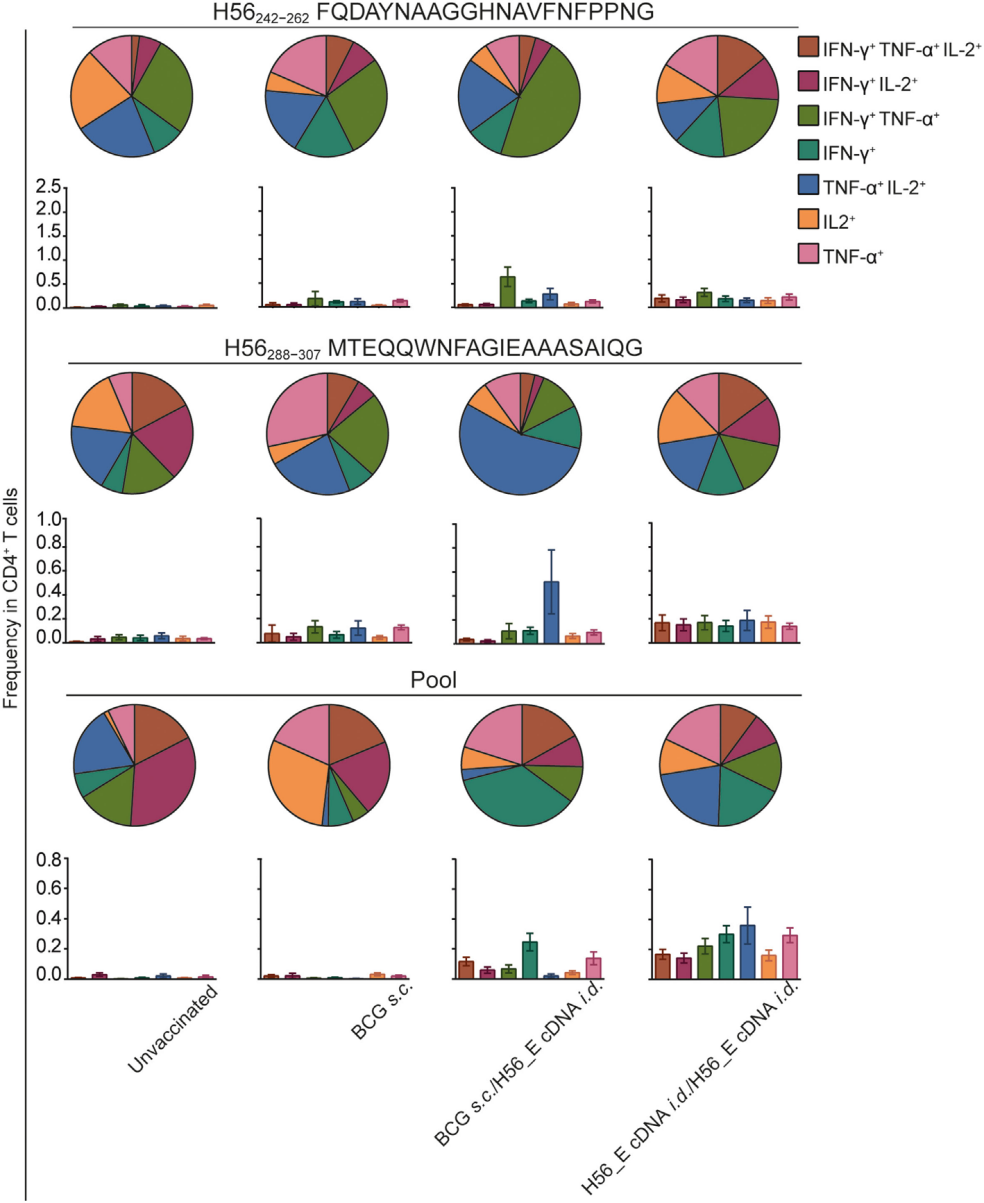
Frequencies in double or triple cytokine positive CD4^+^ T cells is correlated to immunization strategy. Intracellular cytokine staining was performed on splenocytes harvested at day 14 after the booster vaccination and re-stimulated with different H56 peptides. The peptide pools consisted of eighty-five 15-mer peptides spanning the entire sequence of H56, as a measure of the total percentage of H56-antigen-specific cells. CD4^+^ T cell epitopes included H56_242–262_ (Ag85B derived) and H56_288–307_ (ESAT-6 derived). Analysis was done using FACS. The results show pooled data from two independent experiments (*n* = 10 in total). Within the CD4^+^ population, frequencies of peptide-specific single, double, or triple positive cells is shown in bar graphs. The pie graphs denote the proportion of each cytokine-producing subset of the responding cells.

Frequencies of H56-specific multi-cytokine-producing CD4^+^ T cells after different vaccination regimes were analyzed (Figure 5), since cytokine-producing capacity has been correlated to differentiation state (40, 54, 55). BCG induced much lower frequencies of H56-specific cytokine-producing cells overall. Strikingly, there were differences in cytokine production profiles between groups receiving BCG alone, heterologous prime-boost with BCG and H56_E cDNA, or homologous H56_E cDNA prime-boost. Specifically, in the heterologous prime-boosted group, CD4^+^ T cells responding to Ag85B-derived H56_242–262_ were primarily IFN-γ^+^TNF-α^+^, while in the H56_E/H56_E cDNA vaccinated groups they expressed a more mixed cytokine-producing profile. By contrast, CD4^+^ T cells responding to ESAT-6-derived H56_288–307_ were TNF-α^+^IL-2^+^ in the BCG/H56_E group and had mixed profiles in the H56_E/H56_E and the BCG-only vaccinated groups. Responses to the peptide pool were different again, with BCG prime/DNA boost mice showing mostly IFN-γ^+^ cells, while the H56_E/H56_E vaccinated group had a mixed profile.

**Figure 6:**
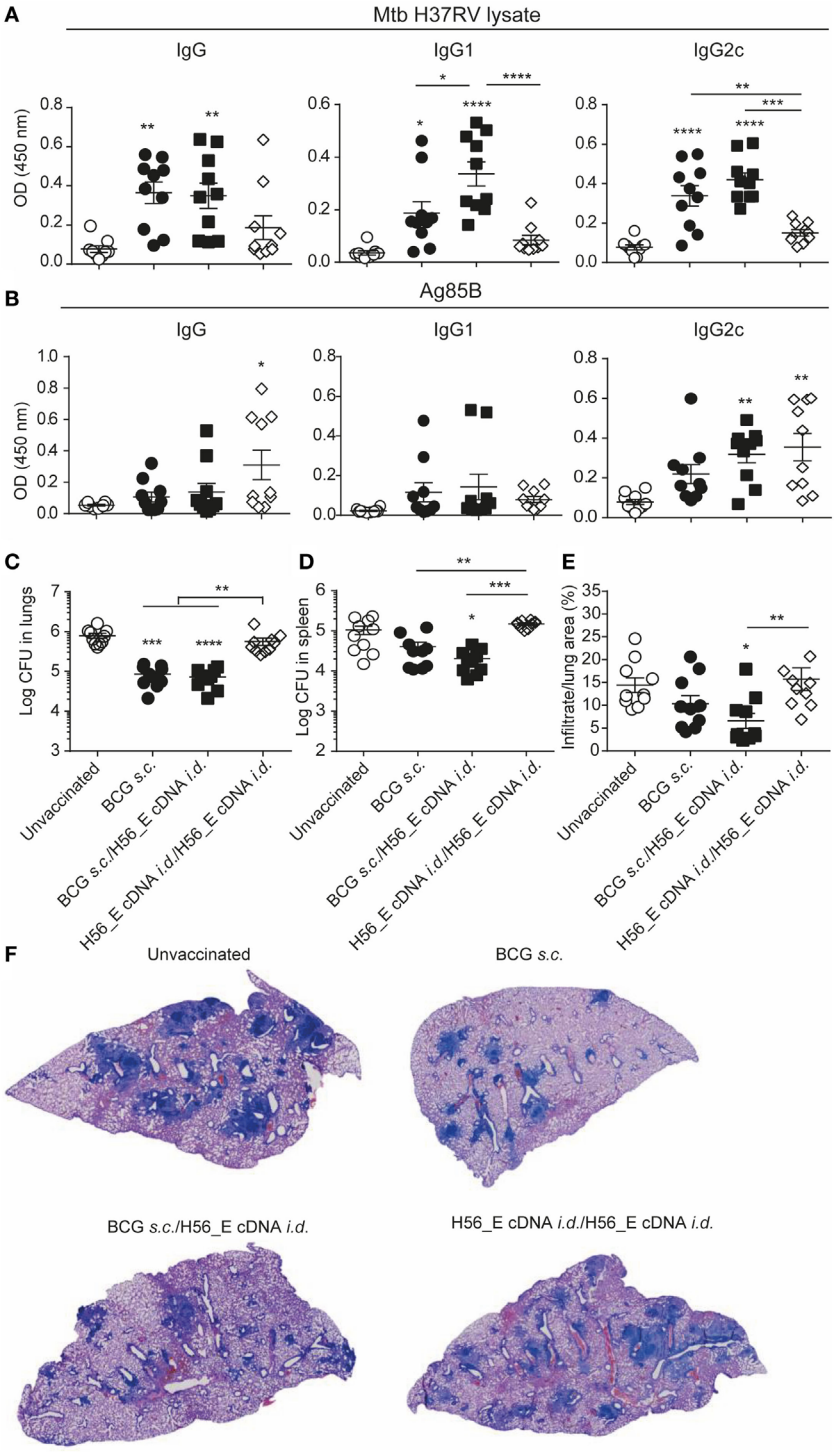
Heterologous prime-boost with BCG/H56_E cDNA decreases lung pathology but not bacterial loads. Serum was harvested at day 14 after the booster vaccination and lungs and spleens were collected at day 90 post *Mycobacterium tuberculosis* (*Mtb*) challenge were plated in serial dilution. Pooled data of two experiments with (*n* = 10 in total per group) are shown ±SEM. **(A,B)** Total IgG, IgG1, and IgG2c antibodies recognizing **(A)** *Mtb* lysate or **(B)** Ag85B were measured in serum of immunized mice at day 40 (total IgG: 1 in 100 dilution, IgG1 and IgG2c, 1 in 10 dilution). **(C,D)** Lungs and spleens collected at day 90 post *Mtb* challenge were plated in serial dilution. Pooled data of two experiments with (*n* = 10 in total per group) is shown ±SEM. **(C)** Bacterial loads in lungs and **(D)** spleen. **(E)** Quantification of percentage of infiltrated area per lung. **(F)** Giemsa staining of infected lungs. Representative histology slides per group are shown. **(A–E)** Significant differences between treatment groups and the unvaccinated group are marked as * and significant differences between groups with lines. *p < 0.05; **p < 0.01; ***p < 0.001; ****p < 0.0001. **(A–E)** ANOVA with Tukey’s multiple comparison test; **(C,D)** Kruskal–Wallis with Dunn’s multiple comparison test.

No significant differences were found in lymph node GC and plasma B cells between groups (data not shown), and antibody levels were also similar (Figures 6A,B) although the anti-mycobacteria IgG1 titer was increased in mice receiving a booster immunization with H56_E cDNA compared to the group vaccinated with BCG alone (Figure 6A). Titers of anti-mycobacteria antibodies in H56_E cDNA/H56_E cDNA prime-boost mice remained as low as in unvaccinated controls (Figure 6A), but Ag85B-specific IgG2c titers were raised (Figure 6B).

### Despite Inducing H56-Specific Immune Responses, a Booster Immunization with H56_E cDNA Does Not Ameliorate Bacterial Loads Compared to BCG Vaccination in Mice

To determine the protective efficacy of the different prime-boost strategies, bacterial loads, and lung pathology were measured 90 days after *Mtb* challenge. BCG and BCG/H56_E cDNA immunized groups had reduced lung bacterial burdens compared to the unvaccinated mice; however, there was no significant improvement in bacterial loads after boosting with H56_E cDNA (Figures 6C,D). Boosting H56_E cDNA prime with a homologous immunization did not increase efficacy, and mice were not protected over unvaccinated controls. Bacterial loads in spleens followed a similar pattern (Figure 6D), although vaccination was not as protective as in the lung. Quantification of infiltrating cells in the lungs by image analysis also revealed H56_E homologous prime-boost mice to be the least protected (Figures 6E,F). Mice primed with BCG and boosted with H56_E cDNA were the only group to show significant improvement in lung pathology compared to the unvaccinated group and tended to have less cell infiltration than the BCG-vaccinated mice too, suggesting that the H56-specific T cell responses associated with boosting with H56_E cDNA did have a beneficial effect in ameliorating disease. Overall, protective efficacy relied on the presence of BCG in the vaccine regimen, suggesting that broader anti-mycobacterial responses may be required for optimal protection in addition to the generation of highly specific responses against particular antigens.

## Discussion

Previously, we demonstrated that the immunogenicity of a DNA vector containing H56 cDNA could be enhanced by fusion of the H56 sequence to cDNA of TTFC and by altering epitope flanking residues to facilitate epitope processing (43). Here, we tested the efficacy of immunization with the optimized constructs against *Mtb* challenge. The results demonstrate that *i.d*. tattoo vaccination with an optimized H56_E cDNA sequence fused to TTFC cDNA, either as a standalone vaccine or as a booster to BCG, did not reduce *Mtb* burdens in the lung compared to BCG alone, although it showed a tendency to improve lung pathology.

Bacillus Calmette–Guérin is the only licensed vaccine against TB, but it provides variable and inadequate protection against the disease (10). Therefore, recombinant BCG vaccines and other live-attenuated mycobacterial strains are being tested as replacement vaccines, while subunit vaccines containing new antigens, adjuvants, or viral vectors are being investigated for their ability to boost BCG (13–18, 40–42). Boosting of BCG prime vaccination with CAF01-adjuvanted *Mtb* fusion protein H56 (containing Ag85B, ESAT-6, and Rv2660 components) increased BCG-induced protection against TB in a murine model (33) and H56 administered in IC31 adjuvant as a booster following BCG prime vaccination was more protective than BCG alone in macaques (42). In order to test alternative methods of subunit vaccine delivery that could be used in prime-boost regimens without the need for adjuvants, we generated H56-encoding DNA constructs with H56 cDNA fused to TTFC cDNA (43). Several naked DNA vaccine candidates have been tested against TB in mice, with various degrees of immunogenicity and prophylactic efficacy (56), but the protective efficacy of tattoo immunization with constructs containing *Mtb* antigens has not been assessed previously. We tested whether *i.d*. H56 cDNA tattoo immunization was protective against TB in a mouse model, either as a standalone vaccine or as a heterologous boost to BCG prime. We found that *i.d*. tattoo immunization with H56 constructs did not protect against TB in mice, although it increased H56-specific T cell responses, spleen GC B cells, and antibody titers when given as a boost to BCG prime. Overall, our results showed that BCG immunization was more protective than the highly specific response to a few antigens induced by *i.d*. H56 cDNA immunization.

Understanding the underlying mechanisms of protective immunity to *Mtb* will assist in the rational design of effective vaccines (57). Our experiments comparing *i.d*. DNA immunization with H56 constructs and *s.c*. vaccination with BCG demonstrated that the two types of vaccines elicited distinct immune responses. BCG induced increased frequencies of CD4^+^ T_EM_, T_FH_, GC B cells, and mycobacteria-specific antibodies, while H56 cDNA immunization induced H56-specific CD4^+^IFN-γ^+^ T cell responses and Ag85B-specific IgG antibodies. BCG shares numerous mycobacterial antigens with *Mtb*, including the immunodominant antigen Ag85B, while the DNA tattoo immunization elicits specific responses against H56, demonstrated by responses against ESAT-6 and Ag85B. Mice vaccinated with BCG accordingly had relatively high levels of mycobacteria-specific antibodies; while mice administered cDNA *i.d*. (particularly the unmodified H56 sequence) developed higher titers of Ag85B-specific antibodies. Both BCG and the H56 cDNA constructs elicited Ag85B-specific CD4^+^ and CD8^+^ T cells, with the peptide H56_242–262_ being a particularly strong inducer of IFN-γ responses by CD4^+^ T cells.

Mice immunized with BCG *s.c*. were better protected against *Mtb* challenge than mice that received H56 cDNA *i.d*., suggesting that broader anti-mycobacterial responses are an important component of vaccine-induced protection. Unlike viruses, which contain only a small number of antigens forming obvious vaccine candidates, bacterial pathogens such as *Mtb* contain thousands of potential antigens, and no single antigen has been identified that is clearly associated with protective responses. This may be why it has been difficult for subunit vaccines to surpass live whole cell vaccines such as BCG in mouse models (10). Live BCG persists in mice for up to 16 months post vaccination and disseminates in the host, with T cell responses peaking around week 32 and waning thereafter (58, 59), whereas T cell responses against DNA vaccines could be measured only up to day 50 (43). Previously, we showed that BCG disseminates to the lungs after *s.c*. vaccination, where it persists for about 55 days (22). *I.d*. tattoo vaccination with BCG showed a trend toward decreased control of lung bacterial burdens compared to *s.c*. vaccination, however, lung pathology and spleen bacterial burdens were similar to that of *s.c*. vaccinated mice. BCG is most commonly administered by *i.d*. injection (not tattoo) in the clinic, and this route was found to be equivalent to percutaneous administration in both efficacy and safety over a 2-year follow-up period (60).

H56 and H56_E cDNA were not protective as standalone vaccines; hence, we tested whether a homologous prime-boost regimen could improve the efficacy, using H56_E cDNA. Homologous H56_E cDNA prime-boost mice were also not protected compared to unvaccinated mice after *Mtb* challenge. These mice had the most vigorous H56-specific CD8^+^ T cell responses, indicating that this response was not sufficiently effective in preventing murine TB, at least during active infection. Furthermore, mice in this group hardly produced any *Mtb*- and Ag85B-specific IgG, with only a few animals producing Ag85B-specific IgG2c. It is possible that alterations to the H56_E sequence may have led to a change in B cell Ag85B epitopes or skewing of immune responses away from Ag85B antibody-producing conditions, since in the single-dose vaccination experiments, H56 cDNA also led to increased antibody titers compared to H56_E cDNA.

Which immune responses are required for optimal protection against TB remains a key question in the attempt to generate a more effective vaccine. In our previous studies with BCG *ΔureC::hly* and BCG *ΔureC::hly ΔnuoG*, increased protection after vaccination against TB coincided with increased T_EM_, T_CM_, T_FH_, GC B cells, and mycobacteria-specific antibody titers as compared to BCG vaccination (22). CD4^+^ T_CM_, by virtue of their capacity to generate new CD4^+^ T_EM_, are considered important in long-term immunity to *Mtb*. Here, boosting BCG by *i.d*. immunization with H56_E cDNA did not induce elevated CD4^+^ T_CM_ over BCG alone. The adjuvant CAF01 induces T_CM_ responses (40) and homing of cytokine-producing cells to the lung parenchyma (61), which could explain why boosting with H56 protein in CAF01 adjuvant was more effective than boosting with the H56 cDNA tattoo immunization. In macaques vaccinated with BCG followed by an H56 booster in IC31 adjuvant, early recall responses to the vaccine antigens were associated with protection (42). A comparison between H56 cDNA and H56 protein/adjuvant immunization could provide further insights into the type of immunity required for protection against TB. In addition, increased H56-specific CD8^+^ T cell responses did not coincide with increased protective efficacy in our model. CD8^+^ T cells play a critical role in vaccine-induced immunity to TB in macaques, whose CD8^+^ T cells are more similar to humans (62). In mice, CD8^+^ T cells appear to play a more important role in the chronic phase of TB than in the acute phase (28). Several studies have shown that antibodies and B cells may also play a role in immunity to TB (51–53). Interestingly, a previous study in guinea pigs found that boosting a recombinant BCG expressing ESAT-6 with intramuscular ESAT-6 cDNA injection reduced protection compared to BCG alone, despite inducing increased antigen-specific IFN-γ responses (63). Together with our study, this suggests that cDNA immunization as a whole might not induce the type of immune responses required for immunity to TB.

In summary, *i.d*. DNA tattoo vaccination using H56 and H56_E constructs alone or in combination with BCG did not significantly improve upon protection induced by BCG vaccination alone in our model, despite inducing strong H56-specific CD4^+^ T cell and CD8^+^ T cell responses. Nevertheless, the H56-specific responses induced by H56_E cDNA vaccination did ameliorate lung pathology, demonstrating the value of adding subunit vaccines containing defined *Mtb* antigens as a booster to BCG. Differences in T cell cytokines and antibody production following the different vaccination regimens as well as the limited number of antigens in H56 compared to BCG may explain the findings. Understanding why the types of immune responses generated by DNA tattoo vaccination does not suffice for protection against TB in contrast to responses generated by BCG or H56 administered with adjuvants such as CAF01 and IC31 will lead to optimized TB vaccination strategies.

## Ethics Statement

This study was carried out in accordance with the recommendations of the GV-SOLAS. The protocol was approved by the State Office for Health and Social Services, Berlin, Germany.

## Author Contributions

AP and NN contributed equally to this work. AP, NN, AS, and SK conceptualized the study and wrote the manuscript. AP, NN, UZ, and SS performed experiments. AP, NN, TD, and VB performed data analysis. All authors contributed to the manuscript preparation. AS and SK are joint senior authors.

## Conflict of Interest Statement

SK is co-inventor/patent holder of BCG *ΔureC::hly* (VPM1002). The other authors declare to have no conflicts of interest.

## Abbreviations

BCG, *Mycobacterium bovis* bacillus Calmette–Guérin; GC, germinal center; *i.d*., intradermal; *Mtb, Mycobacterium tuberculosis*; *s.c*., subcutaneous; TB, tuberculosis; T_CM_, central memory T cells; T_EM_, effector memory T cells; T_FH_, follicular helper T cells; TTFC, tetanus toxin fragment C domain 1.
