# Supplementary material for: Efficacy Testing of H56 cDNA Tattoo Immunization against Tuberculosis in a Mouse Model
Source: Front Immunol. 2017 Dec 11;8:1744. doi: 10.3389/fimmu.2017.01744 (PMC5732355; doi:10.3389/fimmu.2017.01744)
Supplement: Supplementary file 1 [file Table_1.docx]

***Supplementary Materials***

**Efficacy testing of H56 cDNA tattoo immunization against tuberculosis in a mouse model**

Anouk C.M. Platteel^1,2,*^, Natalie Nieuwenhuizen^2,*^, Teresa Domaszewska^2^, Stefanie Schürer^2^, Ulrike Zedler^2^, Volker Brinkmann^3^, Alice J.A.M. Sijts^1,#^ and Stefan H.E. Kaufmann^2,#^

^1^ Department of Infectious Diseases and Immunology, Faculty of Veterinary Medicine, Utrecht University, Utrecht, The Netherlands.

^2^ Department of Immunology, Max Planck Institute for Infection Biology, Berlin, Germany.

^3^ Microscopy Core Facility, Max Planck Institute for Infection Biology, Berlin, Germany

* These authors contributed equally to this work

# Equal senior co-authors

Corresponding author: Stefan H.E. Kaufmann (kaufmann@mpiib-berlin.mpg.de)

**Supplementary Table 1. Statistical differences in peptide specific T cell responses measured by FACS after immunization.** The table presents p-values calculated for differences in cytokine frequencies after stimulation with H56 peptide pools, CD4 epitopes or CD8 epitopes in comparison to unvaccinated or BCG *s.c.* vaccinated mice. Ag85B- and ESAT-6-specific epitopes are distinguished in the table. The p-values for particular cytokine/peptide combinations are calculated using linear models created with cytokine frequency as the dependent variable and treatment as the predictor and corrected for multiple testing with Benjamini-Hochberg method. Significant differences are highlighted in green. The heatmaps are shown in Fig. 1C-D.

| CD4^+^ T cells |  | Compared to BCG *s.c.* | Unvaccinated | BCG *s.c.* | BCG *i.d.* | H56 cDNA *i.d.* | H56_E cDNA *i.d.* |
| --- | --- | --- | --- | --- | --- | --- | --- |
|  | IFN-γ | All peptides incl. pool | No significant differences | | | | |
|  | IL-17 | All peptides incl. pool | No significant differences | | | | |
|  | IL-2 | All peptides incl. pool | No significant differences | | | | |
|  | TNF-α | All peptides incl. pool | No significant differences | | | | |
|  |  |  |  |  |  |  |  |
|  |  | Compared to Unvaccinated | Unvacci-  nated | BCG *s.c.* | BCG *i.d.* | H56 cDNA *i.d.* | H56_E cDNA *i.d.* |
|  | IFN-γ | H56 peptide pool | X | 0,566 | 0,284 | **0,050** | **0,007** |
|  |  | H56_242-262_ (Ag85B) | X | **0,021** | 0,108 | **0,007** | 0,070 |
|  |  | H56_288-307_ (ESAT-6) | X | 0,166 | 0,075 | **0,007** | 0,104 |
|  | IL-17 | All peptides incl. pool | No significant differences | | | | |
|  | IL-2 | All peptides incl. pool | No significant differences | | | | |
|  | TNF-α | All peptides incl. pool | No significant differences | | | | |
|  |  |  |  |  |  |  |  |
| CD8^+^ T cells |  | Compared to BCG *s.c.* | Unvacci-  nated | BCG *s.c.* | BCG *i.d.* | H56 cDNA *i.d.* | H56_E cDNA *i.d.* |
|  | IFN-γ | All peptides incl. pool | No significant differences | | | | |
|  |  |  |  |  |  |  |  |
|  |  | Compared to Unvaccinated | Unvacci-  nated | BCG *s.c.* | BCG *i.d.* | H56 cDNA *i.d.* | H56_E cDNA *i.d.* |
|  | IFN-γ | All peptides incl. pool | No significant differences | | | | |
