# Supplementary material for: Efficacy Testing of H56 cDNA Tattoo Immunization against Tuberculosis in a Mouse Model
Source: Front Immunol. 2017 Dec 11;8:1744. doi: 10.3389/fimmu.2017.01744 (PMC5732355; doi:10.3389/fimmu.2017.01744)
Supplement: Supplementary file 2 [file Table_2.docx]

***Supplementary Materials***

**Efficacy testing of H56 cDNA tattoo immunization against tuberculosis in a mouse model**

Anouk C.M. Platteel^1,2,*^, Natalie Nieuwenhuizen^2,*^, Teresa Domaszewska^2^, Stefanie Schürer^2^, Ulrike Zedler^2^, Volker Brinkmann^3^, Alice J.A.M. Sijts^1,#^ and Stefan H.E. Kaufmann^2,#^

^1^ Department of Infectious Diseases and Immunology, Faculty of Veterinary Medicine, Utrecht University, Utrecht, The Netherlands.

^2^ Department of Immunology, Max Planck Institute for Infection Biology, Berlin, Germany.

^3^ Microscopy Core Facility, Max Planck Institute for Infection Biology, Berlin, Germany

* These authors contributed equally to this work

# Equal senior co-authors

Corresponding author: Stefan H.E. Kaufmann (kaufmann@mpiib-berlin.mpg.de)

**Supplementary Table 2. Statistical differences in peptide specific responses measured by ELISpot after immunization.** The table presents p-values calculated for differences in IFN-γ producing cells after re-stimulation with H56 peptide pool, CD8 epitopes or CD4 epitopes in comparison to unvaccinated or BCG *s.c.* vaccinated mice. The epitopes are distinguished in the table by “CD4”, “CD8”, “Ag85B” and “ESAT-6”. The p-values for particular cytokine/peptide combinations were calculated by linear models created with cytokine frequency as dependent variable and treatment as predictor and corrected for multiple testing with Benjamini-Hochberg method. Significant differences are highlighted in green. The heatmaps are shown in Fig. 1E.

| IFN-γ | Compared to BCG *s.c* | Unvaccinated | BCG *s.c.* | BCG *i.d.* | H56 cDNA *i.d.* | H56_E cDNA *i.d.* |
| --- | --- | --- | --- | --- | --- | --- |
|  | H56 peptide pool | 0,877 | X | 0,562 | **3,38E-06** | 0,136 |
|  |  |  |  |  |  |  |
|  | H56_62-70_ (CD8, Ag85B) | 0,922 | X | 0,517 | 0,851 | 0,922 |
|  | H56_72-80_ (CD8, Ag85B) | 0,851 | X | 0,154 | 0,517 | 0,851 |
|  | H56_95-103_ (CD8, Ag85B) | 0,950 | X | 0,626 | 0,051 | 0,922 |
|  | H56_146-154_ (CD8, Ag85B | 0,922 | X | **0,047** | 0,517 | 0,851 |
|  | H56_161-169_ (CD8, Ag85B) | 0,936 | X | 0,824 | 0,910 | 0,936 |
|  | H56_354-363_ (CD8, ESAT-6) | 0,950 | X | 0,851 | **0,047** | 0,824 |
|  |  |  |  |  |  |  |
|  | H56_242-262_ (CD4, Ag85B) | 0,796 | X | 0,734 | 0,331 | 0,817 |
|  | H56_288-307_ (CD4, ESAT-6) | 0,609 | X | 0,331 | 0,588 | 0,796 |
|  |  |  |  |  |  |  |
| IFN-γ | Compared to Unvaccinated | Unvacci-  nated | BCG *s.c.* | BCG *i.d.* | H56 cDNA *i.d.* | H56_E cDNA *i.d.* |
|  | H56 peptide pool | X | 0,877 | 0,678 | **8,53E-06** | 0,189 |
|  |  |  |  |  |  |  |
|  | H56_62-70_ (CD8, Ag85B) | X | 0,959 | 0,252 | 0,656 | 0,959 |
|  | H56_72-80_ (CD8, Ag85B) | X | 0,959 | 0,495 | 0,959 | 0,959 |
|  | H56_95-103_ (CD8, Ag85B) | X | 0,959 | 0,656 | 0,051 | 0,959 |
|  | H56_146-154_ (CD8, Ag85B) | X | 0,959 | 0,051 | 0,656 | 0,959 |
|  | H56_161-169_ (CD8, Ag85B) | X | 0,959 | 0,959 | 0,959 | 0,959 |
|  | H56_354-363_ (CD8, ESAT-6) | X | 0,959 | 0,959 | 0,051 | 0,921 |
|  |  |  |  |  |  |  |
|  | H56_242-262_ (CD4, Ag85B) | X | 0,799 | 0,799 | 0,093 | 0,799 |
|  | H56_288-307_ (CD4, ESAT-6) | X | 0,799 | 0,799 | 0,093 | 0,799 |
