# Supplementary material for: Efficacy Testing of H56 cDNA Tattoo Immunization against Tuberculosis in a Mouse Model
Source: Front Immunol. 2017 Dec 11;8:1744. doi: 10.3389/fimmu.2017.01744 (PMC5732355; doi:10.3389/fimmu.2017.01744)
Supplement: Supplementary file 3 [file Table_3.docx]

***Supplementary Materials***

**Efficacy testing of H56 cDNA tattoo immunization against tuberculosis in a mouse model**

Anouk C.M. Platteel^1,2,*^, Natalie Nieuwenhuizen^2,*^, Teresa Domaszewska^2^, Stefanie Schürer^2^, Ulrike Zedler^2^, Volker Brinkmann^3^, Alice J.A.M. Sijts^1,#^ and Stefan H.E. Kaufmann^2,#^

^1^ Department of Infectious Diseases and Immunology, Faculty of Veterinary Medicine, Utrecht University, Utrecht, The Netherlands.

^2^ Department of Immunology, Max Planck Institute for Infection Biology, Berlin, Germany.

^3^ Microscopy Core Facility, Max Planck Institute for Infection Biology, Berlin, Germany

* These authors contributed equally to this work

# Equal senior co-authors

Corresponding author: Stefan H.E. Kaufmann (kaufmann@mpiib-berlin.mpg.de)

| CD4^+^ T cells |  | Compared to BCG *s.c.* | Unvaccinated | BCG *s.c.* | BCG *s.c*./  H56_E cDNA *i.d.* | H56_E cDNA *i.d*./ H56_E cDNA *i.d.* |
| --- | --- | --- | --- | --- | --- | --- |
|  | IFN-γ | H56 peptide pool | 0,991 | X | **0,007** | **0,001** |
|  |  | H56_242-262_ (Ag85B) | 0,936 | X | 0,760 | 0,299 |
|  |  | H56_288-307_ (ESAT-6) | 0,936 | X | 0,936 | 0,504 |
|  | IL-17 | All peptides incl. pool | No significant differences | | | |
|  | IL-2 | All peptides incl. pool | No significant differences | | | |
|  | TNF-α | H56 peptide pool | 0,936 | X | 0,116 | **1,77E-05** |
|  |  | H56_242-262_ (Ag85B) | 0,143 | X | 0,116 | 0,416 |
|  |  | H56_288-307_ (ESAT-6) | 0,936 | X | 0,936 | 0,936 |
|  |  |  |  |  |  |  |
|  |  | Compared to Unvaccinated | Unvacci-nated | BCG *s.c.* | BCG *s.c*./  H56_E cDNA *i.d.* | H56_E cDNA *i.d*./ H56_E cDNA *i.d.* |
|  | IFN-γ | H56 peptide pool | X | 0,983 | **0,008** | **0,001** |
|  |  | H56_242-262_ (Ag85B) | X | 0,886 | 0,321 | 0,113 |
|  |  | H56_288-307_ (ESAT-6) | X | 0,886 | 0,734 | 0,243 |
|  | IL-17 | All peptides incl. pool | No significant differences | | | |
|  | IL-2 | All peptides incl. pool | No significant differences | | | |
|  | TNF-α | H56 peptide pool | X | 0,838 | **0,031** | **1,96E-06** |
|  |  | H56_242-262_ (Ag85B) | X | 0,113 | **0,031** | 0,113 |
|  |  | H56_288-307_ (ESAT-6) | X | 0,838 | 0,243 | 0,070 |
|  |  |  |  |  |  |  |
| CD8^+^ T cells |  | Compared to BCG *s.c.* | Unvacci-nated | BCG *s.c.* | BCG *s.c*./  H56_E cDNA *i.d.* | H56_E cDNA *i.d*./ H56_E cDNA *i.d.* |
|  | IFN-γ | H56 peptide pool | 0,847 | X | 0,224 | **0,004** |
|  |  | H56_62-70_ (Ag85B) | 0,177 | X | 0,320 | 0,490 |
|  |  | H56_72-80_ (Ag85B) | 0,428 | X | 0,689 | 0,428 |
|  |  | H56_95-103_ (Ag85B) | **0,004** | X | 0,266 | 0,364 |
|  |  | H56_146-154_ (Ag85B) | **0,009** | X | 0,738 | 0,835 |
|  |  | H56_161-169_ (Ag85B) | **0,018** | X | **0,043** | 0,177 |
|  |  | H56_354-363_ (ESAT-6) | 0,457 | X | 0,696 | **0,027** |
|  |  |  |  |  |  |  |
|  |  | Compared to Unvaccinated | Unvacci-nated | BCG *s.c.* | BCG *s.c*./  H56_E cDNA *i.d.* | H56_E cDNA *i.d*./ H56_E cDNA *i.d.* |
|  | IFN-γ | H56 peptide pool | X | 0,888 | 0,176 | **0,006** |
|  |  | H56_62-70_ (Ag85B) | X | 0,176 | 0,787 | 0,502 |
|  |  | H56_72-80_ (Ag85B) | X | 0,485 | 0,797 | 0,133 |
|  |  | H56_95-103_ (Ag85B) | X | **0,007** | 0,076 | **0,048** |
|  |  | H56_146-154_ (Ag85B) | X | **0,014** | **0,026** | **0,019** |
|  |  | H56_161-169_ (Ag85B) | X | **0,021** | 0,784 | 0,358 |
|  |  | H56_354-363_ (ESAT-6) | X | 0,502 | 0,817 | **0,008** |

**Supplementary Table 3. Statistical differences in peptide specific T cell responses measured by FACS after prime-boost vaccination.** The table presents p-values calculated for differences in cytokine frequencies after stimulation with H56 peptide pools, CD4 epitopes or CD8 epitopes in comparison to unvaccinated or BCG *s.c.* vaccinated mice. Ag85B- and ESAT-6-specific epitopes are distinguished in the table. The p-values for particular cytokine/peptide combinations were calculated by linear models created with cytokine frequency as the dependent variable and treatment as the predictor and corrected for multiple testing with Benjamini-Hochberg method. Significant differences are highlighted in green. The heatmaps are shown in Fig. 4B-C.
