# Supplementary material for: Efficacy Testing of H56 cDNA Tattoo Immunization against Tuberculosis in a Mouse Model
Source: Front Immunol. 2017 Dec 11;8:1744. doi: 10.3389/fimmu.2017.01744 (PMC5732355; doi:10.3389/fimmu.2017.01744)
Supplement: Supplementary file 4 [file Table_4.docx]

***Supplementary Materials***

**Efficacy testing of H56 cDNA tattoo immunization against tuberculosis in a mouse model**

Anouk C.M. Platteel^1,2,*^, Natalie Nieuwenhuizen^2,*^, Teresa Domaszewska^2^, Stefanie Schürer^2^, Ulrike Zedler^2^, Volker Brinkmann^3^, Alice J.A.M. Sijts^1,#^ and Stefan H.E. Kaufmann^2,#^

^1^ Department of Infectious Diseases and Immunology, Faculty of Veterinary Medicine, Utrecht University, Utrecht, The Netherlands.

^2^ Department of Immunology, Max Planck Institute for Infection Biology, Berlin, Germany.

^3^ Microscopy Core Facility, Max Planck Institute for Infection Biology, Berlin, Germany

* These authors contributed equally to this work

# Equal senior co-authors

Corresponding author: Stefan H.E. Kaufmann (kaufmann@mpiib-berlin.mpg.de)

**Supplementary Table 4. Statistical differences in peptide specific T cell responses measured by ELISpot after prime-boost vaccination.** The table presents p-values calculated for differences in cytokine frequencies after the treatments (column names) in comparison to unvaccinated or BCG *s.c.* vaccinated mice. The epitopes are distinguished in the table by “CD4”, “CD8”, “Ag85B” and “ESAT-6”. The p-values for particular cytokine/peptide combinations were calculated with linear models created with cytokine frequency as the dependent variable and treatment as the predictor and corrected for multiple testing with Benjamini-Hochberg method. Significant differences are highlighted in green. The heatmaps are shown in Fig. 4D.

| IFNγ | Compared to BCG *s.c* | Unvaccinated | BCG *s.c.* | BCG *s.c*./  H56_E cDNA *i.d.* | H56_E cDNA *i.d*./ H56_E cDNA *i.d.* |
| --- | --- | --- | --- | --- | --- |
|  | H56 peptide pool | 0,983 | X | 0,069 | **0,002** |
|  |  |  |  |  |  |
|  | H56_62-70_ (CD8, Ag85B) | 0,889 | X | 0,658 | 0,843 |
|  | H56_72-80_ (CD8, Ag85B) | 0,843 | X | 0,603 | 0,808 |
|  | H56_95-103_ (CD8, Ag85B) | 0,973 | X | 0,889 | 0,889 |
|  | H56_146-154_ (CD8, Ag85B) | 0,889 | X | 0,705 | 0,714 |
|  | H56_161-169_ (CD8, Ag85B) | 0,889 | X | 0,753 | 0,072 |
|  | H56_354-363_ (CD8, ESAT-6) | 0,996 | X | 0,843 | 0,072 |
|  |  |  |  |  |  |
|  | H56_242-262_ (CD4, Ag85B) | 0,846 | X | 0,170 | **1,64E-04** |
|  | H56_288-307_ (CD4, ESAT-6) | 0,846 | X | 0,170 | **0,015** |
|  |  |  |  |  |  |
| IFNγ | Compared to Unvaccinated | Unvacci-nated | BCG *s.c.* | BCG *s.c*./ H56_E cDNA *i.d.* | H56_E cDNA *i.d*./ H56_E cDNA *i.d.* |
|  | H56 peptide pool | X | 0,983 | 0,085 | **0,004** |
|  |  |  |  |  |  |
|  | H56_62-70_ (CD8, Ag85B) | X | 0,986 | 0,986 | 0,986 |
|  | H56_72-80_ (CD8, Ag85B) | X | 0,986 | 0,986 | 0,986 |
|  | H56_95-103_ (CD8, Ag85B) | X | 0,986 | 0,986 | 0,986 |
|  | H56_146-154_ (CD8, Ag85B) | X | 0,986 | 0,986 | 0,986 |
|  | H56_161-169_ (CD8, Ag85B) | X | 0,986 | 0,986 | 0,199 |
|  | H56_354-363_ (CD8, ESAT-6) | X | 0,996 | 0,986 | 0,199 |
|  |  |  |  |  |  |
|  | H56_242-262_ (CD4, Ag85B) | X | 0,967 | 0,324 | **0,001** |
|  | H56_288-307_ (CD4, ESAT-6) | X | 0,934 | 0,485 | 0,080 |
